# Total Force Kitchen: Exploring Active-Duty Service Member Performance Optimization Through Cooking

**DOI:** 10.1089/jicm.2023.0025

**Published:** 2024-01-12

**Authors:** Katie Kirkpatrick, Carolyn Kleinberger, Josh Kazman, Salvatore Libretto, Courtney Boyd, Patricia A. Deuster

**Affiliations:** ^1^ Consortium for Health and Military Performance, Department of Military and Emergency Medicine, F. Edward Hébert School of Medicine, Uniformed Services University, Bethesda, MD, USA.; ^2^ Henry M. Jackson Foundation for the Advancement of Military Medicine, Bethesda, MD, USA.

**Keywords:** culinary program, military nutrition, nutritional fitness

## Abstract

**Introduction:**

Obesity, overweight, and suboptimal eating habits are threats to U.S. active-duty service member (SM) nutritional fitness. Offering programs that improve diet quality and nutritional status is of high interest to military leaders.

**Methods:**

Total force kitchen (TFK) was developed as a performance-focused multicomponent program centered around culinary skills with education and skill building in key areas of nutrition, physical activity, and mindfulness. This pilot study's objectives were to determine the feasibility and acceptability of the TFK program, to make recommendations for program modification, and to determine impact on behavior, self-efficacy, and health-related outcomes. Participants were single or geographically single active-duty SMs (*n* = 17) who attended the 12-week, 60-h innovative culinary education and performance optimization program at a local United Service Organization facility. A mixed-method approach assessed pre- and post-program metrics, including attrition rates and participant satisfaction.

**Results:**

The TFK program retention rate was 76.5%. All participants were “somewhat satisfied” or “very satisfied” with the overall TFK program. The highest satisfaction was with the cooking-related components. Improvements in other behavioral (*d* = 0.39, 95% confidence interval [CI]: −0.17 to 0.95), self-rated health (*d* = 0.58, 95% CI: −0.02 to 0.16), and anthropometric measures (e.g., body fat percentage: *d* = −0.01, 95% CI: −0.12 to 0.10) were smaller than improvements in cooking attitudes (*d* = 0.66, 95% CI: 0.17 to 1.13) and self-efficacy for techniques (*d* = 1.80, 95% CI: 0.96 to 2.62). Participants reported positive changes in lifestyle related to what they eat and how they prepare their meals. They also highly valued active learning and instructor knowledge and enthusiasm.

**Discussion:**

This multidisciplinary evidence-based program offers ample opportunities for SMs to gain knowledge, build skills, and engage in a supportive community to optimize their performance through cooking. A successful pilot has the potential to leverage resources for the TFK program expanding its reach and impact to the larger military population and nonmilitary communities.

## Introduction

To meet duty demands, the military requires extraordinary physical, psychological, and social strength. The national obesity epidemic is a “threat to the nation's security,” as Americans are increasingly “too fat to fight.”^1–4^ According to 2017–2018 National Health and Nutrition Examination Survey, 42.4% of American adults are obese, compared with 17%–18% of active-duty service members (SMs) in 2020, an increase from 14% in 2014.^5–8^ Overall, obesity is a serious risk factor for negative health outcomes and chronic diseases. Like the general American population, many active-duty personnel report suboptimal intake of key nutrient-dense foods (fruit, vegetables, whole grains, and dairy).^4^

The average Healthy Eating Index score, a measure of diet quality to assess how well a set of foods aligns with 2015–2020 Dietary Guidelines for Americans recommendations, for Americans is 58 of 100.^9^ A similar Healthy Eating Index score of 59.9 was found in study of 492 Army soldiers.^10^ Both population scores indicate inadequate intake of nutrient-dense foods and excess intake of components to consume in moderation. Rittenhouse et al. found that U.S. soldiers had deficits in several nutritional biomarkers and would benefit from improving multiple dietary components.^10^ Low-quality eating patterns impact military training, injury risk, recovery, mission readiness, and fitness, therefore improving dietary quality should lead to better health and performance.^11^

One approach to encourage SM fitness is the Department of Defense's Total Force Fitness framework.^12,13^ In 2010, the Chairman of the Joints Chiefs of Staff established Total Force Fitness as a “methodology for understanding, assessing, and maintaining the fitness of the Armed Forces.”^14^ The fitness domains are as follows: physical, social, psychological, environmental, nutritional, financial, spiritual, and medical and dental preventative.^12^ Overweight and obesity highlight the interplay among the eight domains. Consequently, there is high interest in military-specific programs that improve nutritional fitness.

Given the desire for engaging and effective nutrition education curriculums, the authors explored teaching kitchens. As defined by Teaching Kitchen Collaborative, teaching kitchens “offer education in basic cooking techniques in addition to other self-care topics like enhanced nutrition …” (https://teachingkitchens.org). Teaching kitchens show changes in behaviors, biomarkers, and clinical outcomes across a variety of patient populations and clinical and geographic settings.^15–18^ A systematic review concluded that cooking classes were associated with improved attitudes, self-efficacy, and a healthier dietary intake in both adults and children.^19^ Participation in teaching kitchens resulted improved energy, healthier food selection, elimination of sugary beverages, and greater focus on optimal sleep hygiene.^15,20–24^

In 2014, Samueli Institute conducted a needs assessment at a large joint service military installation to determine local interest in a teaching kitchen program and how to best customize a Harvard T.H. Chan School of Public Health and Culinary Institute of America teaching kitchen curriculum to meet the needs and expectations of the military community.^25^ More than 534 SMs, health care providers, physician assistant students, chaplains, and spouses responded to the survey and one-on-one interviews. The overall program interest was high (average 7.0 of 10), with the highest interest in nutrition, food for better performance, cooking on a budget, and cooking skills.^25^

As a result, Samueli Institute and other experts customized the curriculum for a military audience by using military-specific language and concepts (such as the Total Force Fitness framework) along with military readiness priorities (i.e., nutrient timing around training) to focus on mental and physical performance optimization (instead of health and disease prevention). They modified logistics for a military setting and conducted a 12-week pilot to increase participants' knowledge and skills to improve nutritional choices, increase physical activity, and reduce stress.^15^

Learning from this military pilot, Uniformed Service University staff further revised and updated the curriculum, now known as total force kitchen (TFK), to meet the needs of the targeted military subpopulation, setting, and staffing along with inclusion of the latest nutrition, physical fitness, and mindfulness content and resources. A pilot study was conducted to (1) assess the feasibility and acceptability of a performance optimization-focused TFK program in a military setting; (2) make recommendations for modifying and refining the program in a military setting; and (3) determine TFK's impact on behavior, self-efficacy, and health-related outcomes.

## Materials and Methods

### Study design

This mixed-methods pilot study, approved by the Uniformed Services University of the Health Sciences Institutional Review Board (Protocol No. MEM-91-4122), was conducted at the United Service Organizations (USO) Warrior and Family Center in Bethesda, Maryland. The USO is a congressionally chartered, private organization dedicated to supporting SMs and their families through programs and services. Regular USO programming incorporates cooking classes and demonstrations, making it a collaboration of interest to local USO leadership.

### TFK program description

TFK included 60 h of instruction with all participants over 12 weeks, with twelve 3-h weeknight sessions and six 4-h biweekly weekend ones. Sessions covered culinary (e.g., basic cooking skills and techniques), nutrition (e.g., nutrition for performance, caffeine, meal planning), mind tactics (e.g., mindfulness, yoga, mindful eating, sleep hygiene), and physical fitness (e.g., cardiovascular, strength training, and recovery) (Table 1). Participants were also offered complementary virtual coaching outside of class sessions, which involved partnering with a health coach via phone or instant messaging to set and review health-related goals.

**Table 1.** Teaching Kitchen Program Schedule with Example Topics

Time	Component	Location	Activity	Example
Weeknight schedule				
5:30–5:55 pm	Dinner	Classroom	Participants enjoy chef-prepared meal; discussion	Grilled salmon, mushroom farrotto, Greek salad with balsamic vinaigrette
5:55–6:00 pm	Mind tactics	Classroom	Group practice	Belly breathing
6:00–6:30 pm	Nutrition^a^	Classroom	Education session, may include hands-on activity	Carbohydrates and whole grains
6:30–6:50 pm	Exercise^b^	Classroom	Education session	Stand up, Warm-up
6:50–7:05 pm	Culinary^c^	Classroom^d^	Education session on cooking techniques and terms	Grains and moist cooking techniques, dressings, and leafy greens
7:05–7:35 pm	Culinary	Kitchen	Chef demonstrations	Dressings, mixed green salad Boiling and steaming green veggies, making pilaf and risotto
7:35–8:05 pm	Culinary	Kitchen	Participant hands-on skills practice	Emulsified dressing, mixed green salad, steam broccoli, boil green beans
8:05–8:20 pm	Culinary	Kitchen	Cleanup Tasting	Enjoy tasting demos and hands-on dishes
8:20–8:30 pm	Program	Kitchen	Wrap-up	Reminder about health coaching, complete weekly feedback
Weekend schedule				
9:00–9:15 am	Breakfast	Classroom	Participants enjoy chef-prepared meal	Breakfast burritos, strawberry banana smoothies
9:15–9:45 am	Integrated topic^e^	Classroom	Education session; might include hands-on activity or skill practice	Mindful eating
9:45–10:00 am	Culinary	Kitchen	Chef discussion	Roasting, braising, pan-searing, poaching
10:00–12:15 pm	Culinary	Kitchen	Participant hands-on skills practice	Asian braised chicken, seared salmon, couscous pilaf, cauliflower cream, roasted cauliflower, cucumber and tomato salad
12:15–12:45 pm	Culinary	Kitchen	Cleanup Tasting and discussion	Enjoy tasting demos and hands-on dishes
12:45–1:00 pm	Program	Kitchen	Wrap-up	Reminder about health coaching, complete weekly feedback

^a^ Nutrition topics: Intro to nutrition, Performance Triad, Whole grains and carbs, Fats and oils, Plant-based proteins, Building flavor and sodium, Food sustainability, Seafood, Breakfast and beverages, Meal planning and shopping, Guide to dining out, Final planning banquet.

^b^ Physical activity topics: Intro to exercise, Move more, Stand up, Warm-up, Breathing Exercises, Recovery, Core stability, Yoga, Cardio, HIIT, Resistance training.

^c^ Culinary topics: Culinary basics, Cooking with whole grains, Vegetable cookery and using healthy fats, Cooking with beans and tofu, hearty soups, and one-pot dishes, Animal proteins, Cooking with seafood, Building a better breakfast, Wraps, Snacks and desserts, Dinner mindfulness, Demo of favorite recipes from class.

^d^ Modified during program to move discussion to kitchen to better meet participant and chef needs.

^e^ Integrated topics: Mindful eating, Mindfulness, Mindful movement, Hunger/fullness, Sleep.

Program staff included subject matter expert instructors, USO program staff and volunteers, and rotating active-duty military chefs. Nutrition, physical activity, and mind tactics civilian instructors had degrees in their areas of expertise and experience with military populations. A nutrition instructor also functioned as the culinary coordinator and worked closely with the chefs to confirm logistics and review recipes, ingredients, and grocery lists. USO staff supported classroom and kitchen setup and cleanup and grocery shopping. USO-Bethesda covered costs for kitchen equipment and food, whereas the research team provided instructor and participant manuals (binders, printing).

### Participants and procedures

**FIG. 1.**
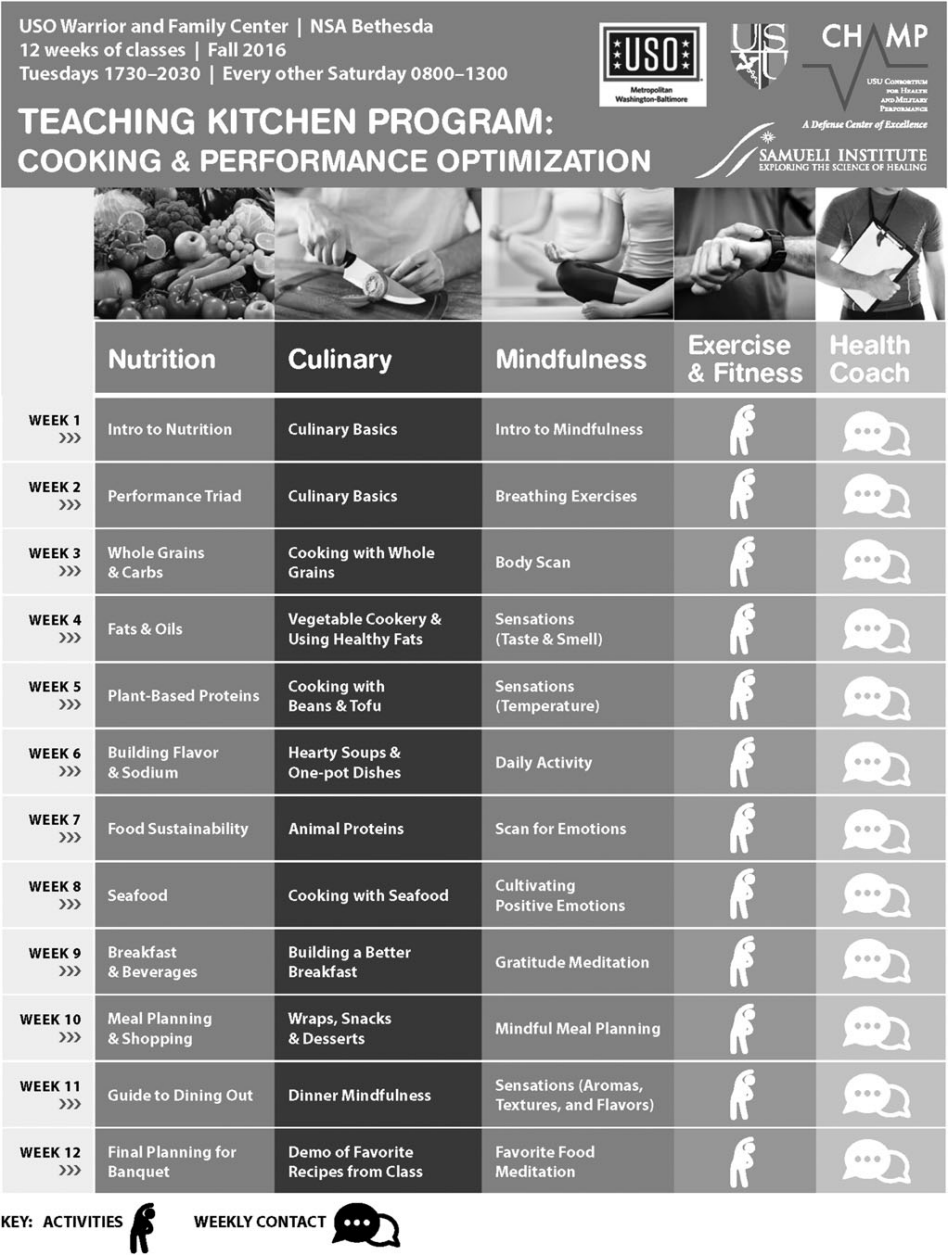
Total force kitchen recruitment flyer.

Participants were primarily recruited over a 6-week period (January to February 2017) through advertising at USO-Bethesda with a table at free lunch and evening events, e-mailing program flyers, and providing program flyers (Fig. 1) to post in local barracks. Interested SMs were assessed for eligibility per predefined criteria: ability to speak and read English and provide consent to participate, active duty, 18–59 years of age, and single or geographically single (those whose families voluntarily do not relocate with them).

Exclusion criteria included: severe food allergies to nuts, gluten, or dairy; certain medical conditions (self-reported diagnosis of cancer, unstable angina, or other cardiovascular condition; prior or planned bariatric surgery; current or planned pregnancy during the study period); military status/assignment (Wounded Warrior Battalion/Soldiers in Transition, medical or nursing student); or self-reported limited ability to understand study procedures, provide informed consent, or fully participate in all program activities (e.g., severe traumatic brain injury or heavy medication). Common reasons for not pursuing the study included inability to make the time commitment (e.g., work or home duties) or program location inconvenient to work/home.

A sample size of 20 participants was chosen based on logistics, space, and staffing. Eligible participants were randomly selected for study inclusion by using a random number generator and stratified for rank, weight status, and gender. Selected individuals were briefed on study procedures, study purpose, and objectives along with potential benefits and risks (minimal risk of cooking-related injury) but were not provided the hypotheses. If interested, they completed a written informed consent before enrollment.

### Data collection

As a pilot study, the primary aim was to examine feasibility and acceptability with a secondary aim to describe changes during the study and generate pre/post-effect size estimates. Primary outcomes included: (1) feasibility, provided by instructor and participant feedback, and (2) acceptability, as measured by attrition rates, and participant satisfaction. Secondary outcomes assessed program impact on (1) cooking and nutritional behavior, attitudes, and self-efficacy and (2) health-related outcomes (i.e., physical activity, sleep, stress, well-being, weight). The statistical analysis plan included qualitative and quantitative approaches utilizing paper-based, validated outcome measures and study-specific surveys along with participant and instructor feedback (Table 2). Some data were electronically collected and maintained in a secure password-protected database; data that required physical collection and storage were maintained in a secure locked office.

**Table 2.** Total Force Kitchen Program Participant Outcome Measures by Time Point

Measure	Eligibility consent and screening	Baseline enrollment, week 1	Program midpoint, week 6	Program end, week 12	1 Month follow-up
Eligibility and Demographics Assessment	X				
Physiological Measurements Form		X	X	X	X
Cooking and Nutrition Assessment		X	X	X	X
Food Frequency Questionnaire–Short GAT		X	X	X	X
Mindful Eating Questionnaire		X	X	X	X
Well-Being Assessment		X	X	X	X
Perceived Stress Scale		X	X	X	X
PSQI		X	X	X	X
PAQ		X	X	X	X
Satisfaction Survey			X	X	
Participant Weekly Feedback Survey		After each session, for total of 16			
Chef Instructor Feedback Questionnaire				X	
Qualitative Interviews				X	
Participant Follow-Up Questionnaire					X
SME and Chef Instructor Follow-Up Questionnaire					X

GAT, Global Assessment Tool; PAQ, Physical Activity Questionnaire; PSQI, Pittsburgh Sleep Quality Index; SME, subject matter expert.

### Feasibility, acceptability, and satisfaction

#### Participant Satisfaction Survey

Thirteen-item survey to capture feedback on the overall program, class topics, and instructors across a 5-point Likert scale, specifically designed for this pilot.

#### Participant Weekly Feedback Survey

Seven-open ended questions completed after each session to document what class material may have surprised, inspired, and/or confused them and any issues.

#### Participant Interviews

At the program's conclusion, nonprogram instructor research team members conducted individual face-to-face structured interviews to assess confidence in using the techniques and tools they learned, class logistics/schedule, program usefulness, and recommendations for future program iterations. Qualitative interviews (∼30 questions, 30 min) were recorded and transcribed for data analysis.

#### Chef Instructor Feedback

Collected informally throughout the program during post-session verbal debriefs on culinary sessions successes and recommended changes, including culinary logistics. When in-person debriefs were not possible, program staff collected feedback via questionnaire.

#### Chef Instructor Feedback Questionnaire

Collected insights regarding overall program flow, program/culinary logistics, and curriculum after the program's conclusion through six-open ended questions.

#### Instructor and Staff Feedback

Collected feedback through a post-program verbal debrief on overall program flow, logistics, and curriculum, including challenges and potential solutions.

### Participant behavior change

#### Culinary and nutrition

Cooking and nutrition questionnaires assessed cooking attitudes, behavior, self-efficacy, and self-efficacy for using basic cooking techniques.^26,27^ The Food Frequency Questionnaire–Short Global Assessment Tool, a 12-item dietary assessment, recorded consumption frequency of certain food categories (e.g., fruit, vegetables, whole grains, dairy).^4^ The Mindful Eating Questionnaire is a 28-item survey across a 4-point Likert scale that measures the construct of mindful eating, a nonjudgmental awareness of physical and emotional sensations associated with eating.^28^

#### Sleep, stress, and physical activity

The 14-item Short Form Health Survey assessed physical and emotional well-being in relation to diet with 3 subscales (i.e., general health, energy/fatigue, emotional well-being) across a 4-point Likert scale.^29,30^ The Perceived Stress Scale, a 10-item survey, assessed the degree to which an individual perceived situations in their life as stressful, measuring events during the last month.^31^ The Pittsburgh Sleep Quality Index, a 19-item questionnaire, assessed sleep quality, latency, duration, and disturbances; habitual sleep efficiency; sleep medication use; and daytime dysfunction.^32^ A Physical Activity Questionnaire assessed weekly exercise patterns by using an 8-item scale.^33^

#### Anthropometric measures

Weight was measured using a metric scale, calibrated to the nearest 0.1 kg, wearing light clothing without shoes. Height was measured using a tape measure, sensitive to the nearest 0.1 cm. Body mass index was then calculated as kg/m^2^. Percent body fat was measured using the InBody720 (BioSpace, Seoul, Korea), an 8-point multifrequency bioelectrical impedance analyzer, while participants wore light clothing without shoes, socks, or metal. Researchers measured neck, waist, and hip circumference horizontally using a flexible, non-stretchable measuring tape, sensitive to the nearest 0.1 cm, by standard techniques per American College of Sports Medicine.

Waist (narrowest point of the torso), hip (largest circumference of the hips), and neck (just below the larynx and above the trapezius muscles) measurements were taken three times, as participants stood up straight with their feet together, head forward, and abdomen and shoulders relaxed. Resting blood pressure and heart rate were measured using the Omron HBP-1300 Professional BP Monitor (Omron, IL) after participants sat in a chair, legs uncrossed, and feet on the floor for 5 min. The cuff size was selected based on arm circumference and placed on the supported and relaxed upper right arm with the artery mark on the cuff in line with the brachial artery.

### Data analysis

Within-group changes from baseline were evaluated at week 6 (midway) for 15 subjects, and after week 12 (post) for 12 subjects. Analyses were primarily descriptive, as is customary for a pilot study.^34^ For within-subject changes, Cohen's *d* was calculated with confidence intervals (CIs) estimated by using Cousineau and Goulet-Pelletier's “lambda-prime” formula.^35^ Values for Cohen's *d* can often be classified as effect size of small (<0.2), medium (∼0.5), or large (>0.8). There was minimal participation at the 1-month follow-up (*n* = 5); therefore, the data were not analyzed. For qualitative data analysis, interviews were transcribed, coded, and then reviewed for common or recurring themes by the research team.

## Results

### Demographics

The TFK program was marketed to Washington, DC metro area military installations. Of the 59 individuals who expressed interest, 21 met the eligibility criteria. Reasons for ineligibility included: Wounded Warrior program participant, married/not single, or inability to attend without family participation. Other individuals did not pursue the program due to inability to make the time commitment (e.g., work or home duties) or program location inconvenient to work/home. Twenty eligible individuals were randomly selected to participate; 17 participants (age = 30.1 ± 9.6, 8 females/9 males) provided written informed consent and were enrolled in the study, which ran from February 28, 2017 to May 20, 2017.

The following includes qualitative and quantitative data analysis by topic.

### Feasibility, acceptability, and satisfaction

Feasibility and acceptability were considered using dropout rate, attendance, program satisfaction scores, and feedback from participant interviews. Four participants dropped out of the program, which resulted in a 76.5% retention rate (attrition rate of 23.5%). This is within the range reported for health behavior change programs generally, and lower than average attrition rates for health behavior change studies, particularly in a military population.^36^ Two participants dropped out early (weeks 1 and 2) while two dropped out late in the program (weeks 10 and 12). All participants shared a reason for withdrawal (i.e., work/school schedule changes, personal/health issues, and the program was not what they hoped for).

Of the 13 participants who completed the program, they attended a median of 13 of 17 total sessions (interquartile range: 11–14.5), including median of 9 for 11 weekday sessions, 4 of 6 weekend sessions. Reasons for absence and tardiness include work responsibilities, medical appointments, out-of-town, traffic, family, or other obligations. Participants shared that time commitment was the most challenging part of TFK; however, 83% said that they would recommend TFK to a peer.

Participants rated their overall program and program component satisfaction at week 6 (midpoint; *n* = 15) and week 12 (program completion; *n* = 11). All participants indicated that they were “somewhat satisfied” or “very satisfied” with the program, stating that the program was “not only teaching me stuff in the kitchen, but teaching me about myself related to the kitchen” and it encouraged them to “stay aware and think about [their] health overall.” Participants also expressed that the program was helpful “especially if you don't have a lot of confidence in the kitchen” and reminded them “healthy food can be delicious and fun.”

The culinary component (hands-on cooking and demonstrations) received the highest satisfaction with 100% of participants feeling “somewhat satisfied” or “very satisfied” at weeks 6 and 12. One participant stated that they “learned how to cook with healthier stuff, that [they] never knew existed” and were motivated to try new foods. One common theme was that chefs made the cooking fun, educational, and entertaining, which contributed to high satisfaction scores. Participants consistently commented on the simplicity and diversity of both recipes and cooking techniques. For example, one participant discussed “a lot of variety in the stuff that we learned … I feel like I've learned the basics in the kitchen.”

Satisfaction for other program components was lower. Moderate improvements were noted for nutrition and mindfulness scores at both the program midpoint and completion. Physical activity and health coaching received the lowest satisfaction scores, with most participants reporting that they felt “somewhat satisfied” and “neutral” about the content, respectively (Table 3).

**Table 3.** Percentage of Total Force Kitchen Participants “Somewhat” or “Very” Satisfied by Program Component (*n* = 15, *n* = 12)

Program component	% Satisfied,*^a^* week 6	% Satisfied,*^a^* week 12	Sample participant quote
Cooking demonstration	100	100	“More confidence in going to cook recipes and coming up with things on my own, or using ingredients that I have vs. looking in my fridge and not knowing what to do. I feel more confident being able to come up with something that will be tasty as well as healthy.”
Hands-on cooking	100	100	“Cutting the vegetables was great! I'm feeling confident and comfortable doing it.”
Nutrition	93	83	“I realized that not everything that I was eating that I thought was healthy, is not really healthy.”
Mindfulness	87	83	“The mindfulness exercise inspired me to do that on a regular basis.”
Exercise	87	58	“I'm going to be leading PT in the morning for my department and I'm definitely going to utilize some of the resources that I learned in class.”
Health coaching	60	50	“Forgot to call.” or “Got too busy.”

^a^ Percentage satisfaction includes responses of “somewhat satisfied” and “very satisfied.”

### Participant behavior change

#### Culinary and nutrition

Improvements, measured by effect size of change, from week 1 to 12 were observed in cooking attitudes, behavior, and self-efficacy (Table 4). Most improvements at week 12 (*n* = 12) were larger than improvements at week 6 (*n* = 15), particularly strong for self-efficacy and self-efficacy of specific techniques.

**Table 4.** Within-Subject Effects of Total Force Kitchen Program Along Cognitive, Well-Being, and Anthropometric Outcomes (*n* = 15, *n* = 12)

Variable by subcategory	Week 6	Week 12
Cooking needs^a^		
Attitude	0.31 (−0.04 to 0.65)	0.66 (0.17 to 1.13)
Behavior	0.25 (−0.10 to 0.59)	0.39 (−0.17 to 0.95)
Self-efficacy	0.61 (0.10 to 1.10)	1.07 (0.42 to 1.69)
Self-efficacy techniques	0.96 (0.44 to 1.47)	1.80 (0.96 to 2.62)
Mindful eating sum score^a^	0.10 (−0.48 to 0.68)	0.48 (−0.14 to 1.10)
Physical activity score^a^	0.07 (−0.55 to 0.69)	−0.07 (−0.48 to 0.35)
Poor sleep quality^b^	−0.16 (−0.40 to 0.08)	−0.27 (−0.76 to 0.23)
Stress^b^	−0.16 (−0.49 to 0.18)	−0.29 (−0.77 to 0.20)
Well-being^a^		
General health	−0.21 (−0.69 to 0.27)^c^	0.58 (−0.02 to 1.16)
Emotional well-being	−0.13 (−0.36 to 0.10)	−0.07 (−0.33 to 0.18)
Energy/fatigue	−0.31 (−0.72 to 0.10)	−0.04 (−0.42 to 0.34)
Anthropometrics^b^		
Percentage body fat	−0.06 (−0.16 to 0.04)	−0.01 (−0.12 to 0.10)
Waist	−0.01 (−0.14 to 0.12)	−0.16 (−0.33 to 0.02)
Hip	0.07 (−0.14 to 0.28)	−0.51 (−0.78 to −0.23)
Waist/hip	−0.06 (−0.20 to 0.08)	0.15 (−0.09 to 0.37)

Measured with Cohen's *d* (95% CI).

^a^ Positive effect sizes indicate improvements over time.

^b^ Negative effect sizes indicate improvements over time.

^c^ One outlier removed due to estimation issues.

CI, confidence interval.

Cooking attitudes, behaviors, and mindful eating improvements were also observed. Cooking attitudes and behavior had a small-to-moderate effect for both week 6 (cooking attitudes: *d* = 0.31, 95% CI: −0.04 to 0.65; behavior: *d* = 0.25, 95% CI: −0.10 to 0.59) and week 12 (cooking attitudes: *d* = 0.66, 95% CI: 0.17 to 1.13; behavior: *d* = 0.39, 95% CI: −0.17 to 0.95), whereas cooking self-efficacy had a moderate-to-large effect at week 6 (self-efficacy: *d* = 0.61, 95% CI: 0.10 to 1.10; self-efficacy for techniques: *d* = 0.96, 95% CI: 0.44 to 1.47) and week 12 (self-efficacy: *d* = 1.07, 95% CI: 0.42 to 1.69; self-efficacy for techniques: *d* = 1.80, 95% CI: 0.96 to 2.62). Mindful eating demonstrated minimal improvement at week 6 (*d* = 0.10, 95% CI: −0.40 to 0.68) but showed a moderate effect at week 12 (*d* = 0.48, 95% CI: −0.14 to 1.10).

#### Sleep, stress, and physical activity

Decreases in poor sleep quality and stress showed improvements with small effects at week 6 (*n* = 15; poor sleep: *d* = −0.16, 95% CI: −0.40 to 0.18) and week 12 (*n* = 12; *d* = −0.27, 95% CI: −0.76 to 0.23). Positive effects were noted along components of well-being (general health, emotional well-being, and energy). At week 6, no improvement of well-being was noted (e.g., general health: *d* = −0.21, 95% CI: −0.69 to 0.27), but week 12 showed a moderate effect improvement (*d* = 0.58, 95% CI: −0.02 to 1.16). No reported significant changes in physical activity at week 6 (*d* = 0.07, 95% CI: −0.55 to 0.69) or week 12 (*d* = −0.07, 95% CI: −0.48 to 0.35).

#### Anthropometric measures

Minimal improvements were noted at either week 6 (e.g., percentage body fat: *d* = −0.06, 95% CI: −0.16 to 0.04; *n* = 15) or week 12 (*d* = −0.01, 95% CI: −0.12 to 0.10; *n* = 12). Hip circumference was the only measure to have a moderate effect at week 12 (*d* = −0.51, 95% CI: −0.78 to −0.23).

## Discussion

This teaching kitchen pilot study targeted single and geographically single SMs as they might be particularly prone to unhealthy nutrition habits (e.g., less likely to cook at home and more likely to seek fast food, takeout, or restaurant food with lower nutrient value) and could benefit from education and community support. TFK nutrition, physical fitness, and mindfulness content was similar to other military fitness and nutrition education programs, but overall provided more in-depth information with a greater emphasis on the interconnectivity of these performance areas. Except for one-time cooking demonstrations or classes, culinary skills and education are often limited for military-supported programs. The TFK program provided a more intensive, hands-on program than traditional military nutrition education efforts with the unique focus on cooking. Only 27% of participants said that they would join TFK without the culinary component, which highlights the great interest in hands-on culinary experiences.

In this population, the TFK program was both feasible and acceptable to promote self-efficacy and confidence and encourage health-related mindset and behaviors. The mixed-method approach was integral to capturing results for this multicomponent program. Participants' improvements were highest with cooking components with some benefits becoming stronger at the program's conclusion. Other behavioral, health, and anthropometric measures improvements were more subtle. This seems appropriate given the relatively short intervention and follow-up and the engaging interactive manner of culinary sessions.

Behavior change improvements aligned with participant satisfaction of program components; culinary components had the highest satisfaction and showed the largest improvements. A 12-week teaching kitchen pilot with active-duty SMs and spouses (*n* = 17) also found significant pre- to post-program improvements in nutritional choices (*p* < 0.0001), attitudes about cooking (*p* = 0.0001), confidence in cooking ability (*p* = 0.0001), confidence in cooking techniques (*p* < 0.0001), and eating with awareness (*p* = 0.0004) along with reductions in waist circumference (*p* = 0.002) and participants who met the criteria for metabolic syndrome (*p* = 0.044).^23^ The average attendance of 81% and an overall very high participant satisfaction are consistent with the results of this TFK pilot.^23^

TFK results are not generalizable to the greater military population given the small sample size and the focus on single/geographically single SMs; however, the program interest and satisfaction are promising. To expand TFK on a larger scale would require considerable logistical support (space, coordination), staffing (volunteers from military installation, local communities, or student programs), and funding (support for food, supplies, and program materials). The research team is hopeful that this might happen in the future.

Importantly, TFK focused on optimal performance, a priority for the target population, to make the content relevant, practical, and meaningful. Participants built and engaged in a community with each other and instructors, which is especially important for single SMs who might have less direct support. Malkawi et al. systematically reviewed military weight management, dietary, and physical activity interventions and “found indications that interventions were most effective when delivered by a trained specialist, utilized theory-based behavior change techniques, promoted both diet and physical activity, and utilized a standardized curriculum.”^36^ The TFK curriculum was delivered by trained specialists, utilized adult learning theory, and promoted diet and physical activity (among other aspects of performance and health). TFK is also an excellent representation of Total Force Fitness framework, which emphasizes understanding and connecting the different fitness domains and the impact of one domain on others.

### Program modifications

Many populations could benefit from a culinary-focused holistic approach to health using the targeted populations' priorities and interests to focus content. Holistic programs such as TFK would benefit from curriculum and program structure modifications to improve feasibility and to maximize satisfaction and participation. Adaptations should remain evidence-based, reflective of current behavior change science, and consider participants' time, skills, knowledge, and interests. Recommended curriculum modifications include more experiential, hands-on activities and content to be more applicable to the target population (e.g., for study population, add practical home-cooking basics, recipes using only basic ingredients, offer performance-focused/advanced physical training exercises). Regardless of modifications, if TFK expands to multiple sites, a standardized curriculum should be used to ensure program consistency and participant experiences, along with the ability to compare analytics from the different sites.

The authors developed a detailed review of specific TFK challenges and potential solutions (Table 5). Due to pilot success and interest, TFK could be expanded or tailored for other military populations and potentially nonmilitary communities. In addition, offering topics such as mind tactics (i.e., mindfulness, meditation, yoga), nutrition (i.e., grocery shopping, culinary skills and tips), or physical activity (i.e., fueling for performance), and multicomponent (i.e., optimize recovery through nutrition, physical activity, mind tactics, and sleep strategies) allow participants further skill building opportunities and familiarize a larger audience with these concepts.

**Table 5.** Total Force Kitchen Program Challenges and Potential Solutions

Program components	Challenges	Potential solutions
Overall program	Absences and tardiness due to length/structure	Offer different program layouts: • “Passport” model that allows participants to attend TFK sessions in nonconsecutive order and across a longer length of time • More weeknight classes with reduced or no weekend sessions • Divide the 12-week curriculum into two 6-week programs (e.g., TFK Basics and TFK Advanced)
	Amount of content presented via classroom presentation	More experiential, hands-on learning
	Late class starts due to tardiness, class feeling rushed	Condense curriculum content
	Absences and tardiness due to work-related and/or other commitments	Require Command sign-off on program participation
Culinary	Insufficient practical home cooking tips (e.g., meal planning, quick meals, preparing multipurpose dishes)	Modify curriculum to align with audience culinary literacy and home-cooking skills; increased focus on healthy meal planning and preparation rather than culinary techniques^a^
	Culinary sessions introduced in the classroom	Plan more hands-on time and cooking demonstrations in the kitchen rather than presentation slides^a^
	Suboptimal cooking demo visibility	Adjust kitchen setup and equipment (e.g., lighting, location, and number of cooking stations)
	USO staff had difficulty interpreting grocery lists and finding specialty items for recipes	Chefs responsible for grocery shopping
Nutrition	Lectures were too long, requested more activity-based learning	Adjust time for more hands-on, experiential activities and questions, reduced nutrition content or topics tailored to participants' specific interests
	Need to integrate cooking skills and nutritional knowledge	Increase chef-nutrition SME collaboration
Physical activity	Session length and frequency	Offer brief sessions that introduce students to accurate, reliable, evidence-based physical activity information and guidance
	Curriculum too basic for active-duty military	Offer either a performance-focused curriculum or yoga sequence tailored to active-duty military
Mind tactics	Insufficient time to dive deeply into content, which was presented at class start and sometimes missed due to tardiness	Reduce length of less popular (e.g., physical activity) components to spend more time on mind tactics; offer a focused mind tactics program
Health coaching	Underutilized resource as it was participant-directed outside of TFK sessions	Include participant personal goals within TFK sessions: allow time for instructor(s) to answer content-related questions; opportunity for participants to discuss specific individual goals with knowledgeable instructors Offer virtual health coaching as an optional resource

^a^ Adjustments made during the TFK program.

SME, subject matter expert; TFK, total force kitchen.

### Limitations

The pilot did not include a control group for comparison as it was designed as a pilot pre–post study. The study's eligibility criteria were limited to single and geographically single active-duty SM and due to time constraints, participants were only recruited over 6 weeks. It is likely the number of interested and enrolled participants would have been larger with less stringent eligibility criteria and a longer recruitment phase. There is a potential for selection bias given the limited recruitment time and strategies, but the researchers attempted to limit social desirability bias by utilizing anonymous feedback and surveys and nonprogram research team members to conduct end of program interviews. Additionally, the current intervention and/or follow-up period might not have been long enough to see health- and behavior-related changes. Finally, the authors calculated effect sizes and CIs to convey mean within-group changes and their variability. Due to the small sample size and limitations, these results should not be used to make inferences or generalize to the average military population.

## Conclusions

The knowledge, skills, understanding, and confidence of how to prepare and choose the right fuel is a challenge for many SMs. The multidisciplinary, evidence-based approach of TFK offers ample opportunities to gain knowledge, build skills, and engage in a supportive community through a fun, engaging cooking platform. The education and resources target key areas and align with the Total Force Fitness framework to promote SM performance and well-being through practical lifestyle changes. With its ability to leverage resources to make the TFK program accessible to a large military community, the USOs potential reach and impact on nutritional fitness are substantial. In addition, similar curriculums could be offered for populations at nutritional risk, such as college students, young adults, and those with health-related concerns.

## Supplementary Material

Supplemental data
