# Supplementary material for: Total Force Kitchen: Exploring Active-Duty Service Member Performance Optimization Through Cooking
Source: J Integr Complement Med. 2024 Jan 12;30(1):66–76. doi: 10.1089/jicm.2023.0025 (PMC10801678; doi:10.1089/jicm.2023.0025)
Supplement: Supplemental data [file Suppl_Data.zip › Food_Frequency_Questionnaire (1).pdf]

Subject ID: | | | | | | |

Date: | | | | | | | | |

T:________

Food Frequency Questionnaire - Short GAT

Pilot: Teaching Kitchen at CHAMP/USO Bethesda

Examples (Note: only a few examples of each are listed to remind you of the types of food in each category many more are possible):

FRUIT - fresh, frozen, canned or dried, or 100% fruit juices. A serving is 1 cup of fruit, or 1/2 cup of fruit juice

VEGETABLES - fresh, frozen, canned, cooked or raw: dark green vegetables (broccoli, spinach, most greens), orange vegetables (carrots, sweet potatoes, winter squash, pumpkin), legumes (dry beans, chick peas, tofu), starchy vegetables (corn, white potatoes, green peas), and other (tomatoes, cabbage, celery, cucumber, lettuce, onions, peppers, green beans, cauliflower, mushrooms, summer squash, etc). A serving is 1 cup of raw vegetables, or ½ cup of cooked vegetables

WHOLE GRAINS - rye, whole wheat, or heavily seeded bread, brown or wild rice, whole wheat pasta or crackers, oatmeal, or corn tacos. A serving is 1 slice of bread, or ½ cup grains

DAIRY - regular/ whole fat milk; low or reduced fat milk (2%, 1%, 1/2 % or skim), yogurt, cottage cheese, low fat cheese, frozen low fat yogurt, soy milk or other calcium fortified foods (orange juice, soy/rice milk, breakfast cereals, etc). A serving is 8 ounces of liquid or 1 ounce of cheese.

Items 1-4. Think about the past 30 days. How often did you eat/drink the following foods/beverages?

	4 or More Servings per Day	2 to 3 Servings per Day	1 Serving per Day	3 to 6 Servings per Week	1 or 2 Servings per Week	Rarely or Never	No Answer
1. Fruit	○	○	○	○	○	○	○
2. Vegetables	○	○	○	○	○	○	○
3. Whole Grains	○	○	○	○	○	○	○
4. Dairy	○	○	○	○	○	○	○

Think about the past 30 days. Please choose the appropriate response.

	7 Times per Week	6 Times per Week	4 to 5 Times per Week	2 to 3 Times per Week	Never	No Answer
5. How many times per week do you eat breakfast?	○	○	○	○	○	○

Food Frequency Questionnaire - Short GAT

Pilot: Teaching Kitchen at CHAMP/USO Bethesda

Think about the past 30 days. Please choose the appropriate response.

	8 or More Servings per Day	5 to 7 Servings per Day	2 to 4 Serving per Day	1 Serving per Day	None	No Answer
6. On an average day, how many servings of water do you drink? *(a serving is 8 oz; a cup of water is 8-12 oz; a standard Camelbak or Nalgene bottle has 24 oz or 3 servings).*	○	○	○	○	○	○

	Most of the Time	Often	Sometimes	Rarely	Never	N/A (Do not participate in strenuous exercise sessions)
7. Recovery Fueling: Do you typically consume a healthy snack within 60 minutes after a strenous exercise session? *(Examples include 1 piece of fruit, a handful of nuts, 1 small yogurt container, 1 cup of milk, 1 granola bar, or 1 sports bar)*	○	○	○	○	○	○

	4 or More Times per Week	2 or 3 Times per Week	1 Time per Week	2 Times per Month	1 Time per Month	Rarely or Never	No Answer
8. Fish *(Examples include tuna, salmon, or other non-fried fish)*	○	○	○	○	○	○	○

Subject ID: | | | | | | |

Date: | | | | | | | | |

T:________

Food Frequency Questionnaire - Short GAT

Pilot: Teaching Kitchen at CHAMP/USO Bethesda

Items 9 - 11. Think about the past 7 days.

How many servings of each of the following do you have per week? (1 serving of meat is about the size of a computer mouse.)

9. Red Meat *(Beef, Pork, Lamb, Veal)* _________

10. Poultry *(Chicken, Turkey)* _________

11. Processed meats *(Deli meats, bacon, sausage, hot dogs)* _________

12. Think about the past 7 days. How many servings (about 1 oz) of nuts do you consume per week?

____
