# Supplementary material for: Total Force Kitchen: Exploring Active-Duty Service Member Performance Optimization Through Cooking
Source: J Integr Complement Med. 2024 Jan 12;30(1):66–76. doi: 10.1089/jicm.2023.0025 (PMC10801678; doi:10.1089/jicm.2023.0025)
Supplement: Supplemental data [file Suppl_Data.zip › Mindful Eating Questionnaire.pdf]

Subject ID: | | | | | | |

Date: | | | | | | | | |

T:________

Mindful Eating Questionnaire

Pilot: Teaching Kitchen at CHAMP/USO Bethesda

Please circle the answer that most relates to you.

	Never / Rarely	Sometimes	Often	Usually / Always
1. I eat so quickly that I don't taste what I'm eating.	○	○	○	○
2. When I eat at "all you can eat" buffets, I tend to overeat.	○	○	○	○
3. At a party where there is a lot of good food, I notice when it makes me want to eat more food than I should.	○	○	○	○
4. I recognize when food advertisements make me want to eat.	○	○	○	○
5. When a restaurant portion is too large, I stop eating when I'm full.	○	○	○	○
6. My thoughts tend to wander while I am eating.	○	○	○	○
7. When I'm eating one of my favorite foods, I don't recognize when I've had enough.	○	○	○	○
8. I notice when just going into a movie theater makes me want to eat candy or popcorn.	○	○	○	○
9. If it doesn't cost much more, I get the larger size food or drink regardless of how hungry I feel.	○	○	○	○
10. I notice when there are subtle flavors in the foods I eat.	○	○	○	○
11. If there are leftovers that I like, I take a second helping even though I'm full.	○	○	○	○
12. When eating a pleasant meal, I notice if it makes me feel relaxed.	○	○	○	○
13. I snack without noticing that I am eating.	○	○	○	○
14. When I eat a big meal, I notice if it makes me feel sluggish.	○	○	○	○
15. I stop eating when I'm full even when eating something that I love.	○	○	○	○

Mindful Eating Questionnaire

Pilot: Teaching Kitchen at CHAMP/USO Bethesda

Please circle the answer that most relates to you.

	Never / Rarely	Sometimes	Often	Usually / Always
16. I appreciate the way my food looks on my plate.	○	○	○	○
17. When I'm feeling stressed at work, I'll go find something to eat.	○	○	○	○
18. If there's good food at a party, I'll continue eating even after I'm full.	○	○	○	○
19. When I'm sad, I eat to feel better.	○	○	○	○
20. I notice when foods and drinks are too sweet.	○	○	○	○
21. Before I eat I take a moment to appreciate the colors and smells of my food.	○	○	○	○
22. I taste every bite of food that I eat.	○	○	○	○
23. I recognize when I'm eating and not hungry.	○	○	○	○
24. I notice when I'm eating from a dish of candy just because it's there.	○	○	○	○
25. When I'm at a restaurant, I can tell when the portion I've been served is too large for me.	○	○	○	○
26. I notice when the food I eat affects my emotional state.	○	○	○	○
27. I have trouble not eating ice cream, cookies, or chips if they're around the house.	○	○	○	○
28. I think about things I need to do while I am eating.	○	○	○	○
