# Supplementary material for: Total Force Kitchen: Exploring Active-Duty Service Member Performance Optimization Through Cooking
Source: J Integr Complement Med. 2024 Jan 12;30(1):66–76. doi: 10.1089/jicm.2023.0025 (PMC10801678; doi:10.1089/jicm.2023.0025)
Supplement: Supplemental data [file Suppl_Data.zip › Physical_Activity_Questionnaire.pdf]

Subject ID: [ ][ ][ ][ ][ ][ ]

Date: [ ][ ][ ][ ][ ][ ][ ][ ]

T:________

Physical Activity Questionnaire (PAQ)

Pilot: Teaching Kitchen at CHAMP/USO Bethesda

Physical Activity

We are trying to find out about your level of physical activity from the last 7 days (in the last week). This includes activities that make you sweat, make your legs feel tired, or make you breathe hard, such as team sports, running, strenuous occupational activities, and others.

Remember: There are no right and wrong answers—this is not a test. Please answer all the questions as honestly and accurately as you can—this is very important.

1. Physical activity in your spare time: Have you done any of the following activities in the past 7 days (last week)? If yes, how many times? (Mark only one circle per row).

	No	1-2	3-4	5-6	7 times or more
a. Rock Climbing	○	○	○	○	○
b. Rowing / Canoeing	○	○	○	○	○
c. Tennis / Squash	○	○	○	○	○
d. Stair Climber (or other similar equipment)	○	○	○	○	○
e. Walking for exercise	○	○	○	○	○
f. Heavy Yard Work	○	○	○	○	○
g. Jogging or Running	○	○	○	○	○
h. Bicycling	○	○	○	○	○
i. Aerobics (or other exercise class)	○	○	○	○	○
j. Swimming	○	○	○	○	○
k. Baseball, softball	○	○	○	○	○
l. Dance	○	○	○	○	○
m. Football	○	○	○	○	○
n. Badminton	○	○	○	○	○
o. Soccer	○	○	○	○	○
p. Street / Floor Hockey	○	○	○	○	○
q. Volleyball	○	○	○	○	○
r. Basketball	○	○	○	○	○
s. Skating (in-line / ice)	○	○	○	○	○
t. Cross-country skiing	○	○	○	○	○
u. Ice Hockey / Ringette	○	○	○	○	○
v. Martial Arts	○	○	○	○	○
w. Weight Training	○	○	○	○	○
Other:					
x. ______________________	○	○	○	○	○
y. ______________________	○	○	○	○	○

Physical Activity Questionnaire (PAQ)

Pilot: Teaching Kitchen at CHAMP/USO Bethesda

Physical Activity

We are trying to find out about your level of physical activity from the last 7 days (in the last week). This includes activities that make you sweat, make your legs feel tired, or make you breathe hard, such as team sports, running, strenuous occupational activities, and others.

Remember: There are no right and wrong answers—this is not a test. Please answer all the questions as honestly and accurately as you can—this is very important.

2. In the last 7 days, during the morning, how often were you very active *(for example: playing sports, exercise classes, strenuous occupational activity)? (Check one only.)*

- ◯ None
- ◯ 1 time last week
- ◯ 2 or 3 times last week
- ◯ 4 or 5 times last week
- ◯ 6 or 7 times last week

3. In the last 7 days, after lunch and before supper, how often were you very active *(for example: playing sports, exercise classes, strenuous occupational activity)? (Check one only.)*

- ◯ None
- ◯ 1 time last week
- ◯ 2 or 3 times last week
- ◯ 4 or 5 times last week
- ◯ 6 or 7 times last week

4. In the last 7 days, during the evening, how often were you very active *(for example: playing sports, exercise classes, strenuous occupational activity)? (Check one only.)*

- ◯ None
- ◯ 1 time last week
- ◯ 2 or 3 times last week
- ◯ 4 or 5 times last week
- ◯ 6 or 7 times last week

Subject ID: | | | | | | |

Date: | | | | | | | | |

T:________

Physical Activity Questionnaire (PAQ)

Pilot: Teaching Kitchen at CHAMP/USO Bethesda

Physical Activity

We are trying to find out about your level of physical activity from the last 7 days (in the last week). This includes activities that make you sweat, make your legs feel tired, or make you breathe hard, such as team sports, running, strenuous occupational activities, and others.

Remember: There are no right and wrong answers—this is not a test. Please answer all the questions as honestly and accurately as you can—this is very important.

5. On the last weekend, how often were you very active *(for example: playing sports, exercise classes, strenuous occupational activity)? (Check one only.)*

- ◯ None
- ◯ 1 time
- ◯ 2 or 3 times
- ◯ 4 or 5 times
- ◯ 6 or more times

6. Which one of the following describes you best for the last 7 days? *Read all five statements before deciding on the one answer that describes you.*

- ◯ All or most of my free time was spent doing things that involve little physical effort.
- ◯ I sometimes (1–2 times last week) did physical things in my free time *(e.g. played sports, went running, swimming, bike riding, did aerobics).*
- ◯ I often (3–4 times last week) did physical things in my free time.
- ◯ I quite often (5–6 times last week) did physical things in my free time
- ◯ I very often (7 or more times last week) did physical things in my free time.

7. Mark how often you did physical activity *(for example: playing sports, exercise classes, strenuous occupational activity).*

	None	Little Bit	Medium	Often	Very Often
Monday	◯	◯	◯	◯	◯
Tuesday	◯	◯	◯	◯	◯
Wednesday	◯	◯	◯	◯	◯
Thursday	◯	◯	◯	◯	◯
Friday	◯	◯	◯	◯	◯

8. Were you sick last week, or did anything prevent you from doing your normal physical activities? *(Check one.)*

- ◯ Yes
- ◯ No

If YES, what prevented you? ____

____
