# Supplementary material for: Total Force Kitchen: Exploring Active-Duty Service Member Performance Optimization Through Cooking
Source: J Integr Complement Med. 2024 Jan 12;30(1):66–76. doi: 10.1089/jicm.2023.0025 (PMC10801678; doi:10.1089/jicm.2023.0025)
Supplement: Supplemental data [file Suppl_Data.zip › PSQI.pdf]

Subject ID: | | | | | | |

Date: | | | | | | | | |

T:________

Pittsburgh Sleep Quality Index (PSQI)

Pilot: Teaching Kitchen at CHAMP/USO Bethesda

Instructions: The following questions relate to your usual sleep habits during the past month ONLY. Your answers should indicate the most accurate reply for the majority of days and nights in the past month. Please answer all questions.

1. During the past month, when have you gone to bed at night?
USUAL BED TIME: _____________

2. During the past month, how long (in minutes) has it usually taken you to fall asleep each night?
NUMBER OF MINUTES: _______________

3. During the past month, when have you usually gotten up in the morning?
USUAL GETTING UP TIME: _______________

4. During the past month, how many hours of actual sleep did you get at night? (This may be different than the number of hours you spend in bed.)
HOURS OF SLEEP PER NIGHT: ______________

For each of the remaining questions, check the one best response. Please answer all questions.

5. During the past month, how often have you had trouble sleeping because you...	Not during the past month	Less than once a week	Once or twice a week	Three or more times a week
a. Cannot get sleep within 30 minutes	◯	◯	◯	◯
b. Wake up in the middle of the night or early morning	◯	◯	◯	◯
c. Have to get up to use the bathroom	◯	◯	◯	◯
d. Cannot breathe comfortably	◯	◯	◯	◯
e. Cough or snore loudly	◯	◯	◯	◯
f. Feel too cold	◯	◯	◯	◯
g. Feel too hot	◯	◯	◯	◯
h. Had bad dreams	◯	◯	◯	◯
i. Have pain	◯	◯	◯	◯

j. Other reason(s), please describe: ____
How often during the past month have you had trouble sleeping because of this?

◯ Not during the past month ◯ Less than once a week ◯ Once or twice a week ◯ Three or more times a week

6. During the past month, how would you rate your sleep quality overall?

◯ Very good ◯ Fairly Good ◯ Fairly Bad ◯ Very Bad

7. During the past month, how often have you taken medicine (Prescribed or "over the counter") to help you sleep?

◯ Not during the past month ◯ Less than once a week ◯ Once or twice a week ◯ Three or more times a week

Pittsburgh Sleep Quality Index (PSQI)
Pilot: Teaching Kitchen at CHAMP/USO Bethesda

8. During the past month, how often have you had trouble staying awake while driving, eating meals, or engaging in social activity?

- ○ Not during the past month
- ○ Less than once a week
- ○ Once or twice a week
- ○ Three or more times a week

9. During the past month, how much of a problem has it been for you to keep up with enough enthusiasm to get things done?sleep?

- ○ No Problem at all
- ○ Only a very slight problem
- ○ Somewhat of a problem
- ○ A very big problem

10. Do you have a bed partner or share a room?

- ○ No bed partner or do not share a room
- ○ Partner/ flatmate in other room
- ○ Partner in same room, but not in same bed
- ○ Partner in same bed

11. If you have a bed partner or share a room, ask him/her how in the past month you have had....	Not during the past month	Less than once a week	Once or twice a week	Three or more times a week
a. Loud snoring	○	○	○	○
b. Long pauses between breaths while asleep	○	○	○	○
c. Legs twitching or jerking while you sleep	○	○	○	○
d. Episodes of disorientation or confusion during sleep.	○	○	○	○

e. Other restlessness while you sleep. Please describe: ____________________

- ○ Not during the past month
- ○ Less than once a week
- ○ Once or twice a week
- ○ Three or more times a week
