# Supplementary material for: Total Force Kitchen: Exploring Active-Duty Service Member Performance Optimization Through Cooking
Source: J Integr Complement Med. 2024 Jan 12;30(1):66–76. doi: 10.1089/jicm.2023.0025 (PMC10801678; doi:10.1089/jicm.2023.0025)
Supplement: Supplemental data [file Suppl_Data.zip › PSS.pdf]

Subject ID: | | | | | | |

Date: | | | | | | | | |

T:________

# Perceived Stress Scale

## Pilot: Teaching Kitchen at CHAMP/USO Bethesda

| | Never | Almost Never | Sometimes | Fairly Often | Very Often |
|---|---|---|---|---|---|
| 1. In the last month, how often have you been upset because of something that happened unexpectedly? | ○ | ○ | ○ | ○ | ○ |
| 2. In the last month, how often have you felt that you were unable to control the important things in your life? | ○ | ○ | ○ | ○ | ○ |
| 3. In the last month, how often have you felt nervous and "stressed"? | ○ | ○ | ○ | ○ | ○ |
| 4. In the last month, how often have you felt confident about your ability to handle your personal problems? | ○ | ○ | ○ | ○ | ○ |
| 5. In the last month, how often have you felt that things were going your way? | ○ | ○ | ○ | ○ | ○ |
| 6. In the last month, how often have you found that you could not cope with all the things that you had to do? | ○ | ○ | ○ | ○ | ○ |
| 7. In the last month, how often have you been able to control irritations in your life? | ○ | ○ | ○ | ○ | ○ |
| 8. In the last month, how often have you felt that you were on top of things? | ○ | ○ | ○ | ○ | ○ |
| 9. In the last month, how often have you been angered because of things that were outside of your control? | ○ | ○ | ○ | ○ | ○ |
| 10. In the last month, how often have you felt difficulties were piling up so high that you could not overcome them? | ○ | ○ | ○ | ○ | ○ |
