# Supplementary material for: Total Force Kitchen: Exploring Active-Duty Service Member Performance Optimization Through Cooking
Source: J Integr Complement Med. 2024 Jan 12;30(1):66–76. doi: 10.1089/jicm.2023.0025 (PMC10801678; doi:10.1089/jicm.2023.0025)
Supplement: Supplemental data [file Suppl_Data.zip › Qualitative Interview- TK - updated 05.03.17.pdf]

Qualitative Interview Probes (Revised 05.03.17)

Guide for 30-minute one-on-one qualitative interview at 12-week of TK program

Participant's Behavior Change

1. What were the most impactful changes to your health/lifestyle due to participating in Teaching Kitchen (e.g., weight changes, clothes fitting differently, sleep, stress, etc.)?
2. As a result of this program, are you more confident in:
    a. Your skills and abilities to plan, shop, prepare, and cook meals?
    b. Your skills and abilities to create your own exercise and recovery program?
    c. Your skills and abilities to identify nutritious foods that are supportive of your performance and health goals?
    d. Using breathing and mindfulness techniques to manage stress?

Program Specific Feedback

Program Logistics

3. Were the classes offered on days that were convenient for you to participate? If not, what would be a better alternative?
4. Were the classes offered at convenient times? If not, what would be a better alternative?
5. Were the length of the classes (hrs on Tues/Sat) appropriate for learning and sustaining your attention? If not, what would be a better alternative?
6. Was the length of the program (12 weeks) adequate? If not, what would you recommend?
7. When you missed classes during the 12 week program, what were your reasons for missing (e.g., work, competing priorities, or not interested in the topic for the week, etc.,)?

8. If you could change the overall set-up, to include the schedule and layout of weekly events, what would you do differently?
9. Was the additional Saturday class helpful for gaining confidence and practicing the skills learned during the previous class? Why or why not?
10. What type of follow-up support would be most helpful after completing 12 weeks (e.g., monthly classes, coaching, informal group gatherings, etc.)

Culinary

11. How would you rate the overall quality of the cooking demonstration and instructions? Did you find it educational v. entertaining/novel?
12. What aspects of the USO kitchen kitchen set-up did you find most helpful (e.g., open space) and/or least helpful (e.g., hearing the instructors) during training?
13. Do you feel the hands-on cooking time was sufficient? If not, what length of time would you recommend?
14. Do you feel the culinary techniques built upon each other in a meaningful and helpful way across the 12 weeks?

Nutrition

15. Do you feel there was enough time spent on nutrition education?
16. What topics would you like to learn more about?
17. What topics would you like to have seen least of?

Physical Activity

18. Do you feel there was enough time spent on physical activity?
19. What topics would you like to learn more about?
20. What topics would you like to have seen least of?

Mind Tactics

21. Do you feel there was enough time spent on breathing, mindfulness, sleep optimization, other techniques?
22. What topics would you like to learn more about?
23. What topics would you like to have seen least of?

Program Variations

24. Would you have liked to spend more time or less time in this program, or neither?
25. Would you participate in this program without the culinary component?
26. Would you participate in a program dedicated to only one aspect of the health and wellness component presented in Teaching Kitchen (e.g., nutrition basics or mindfulness techniques)?

General

27. What was the most challenging part of the program?
28. Was there anything you wish was covered in the program that wasn't? Please explain.
29. Would you recommend this program to fellow service members? Why or why not?
30. Please provide any additional suggestions for improving this program that have not previously been covered.
