# Supplementary material for: Total Force Kitchen: Exploring Active-Duty Service Member Performance Optimization Through Cooking
Source: J Integr Complement Med. 2024 Jan 12;30(1):66–76. doi: 10.1089/jicm.2023.0025 (PMC10801678; doi:10.1089/jicm.2023.0025)
Supplement: Supplemental data [file Suppl_Data.zip › Satisfaction Questionnaire.pdf]

Subject ID: ☐☐☐☐☐☐

Date: ☐☐☐☐☐☐☐☐

T:________

Satisfaction of Program

Pilot: Teaching Kitchen at CHAMP/USO Bethesda

1. On a scale of 1 to 5, how would you rate your experience with this program overall? Please circle.

Very Dissatisfied	Somewhat Dissatisfied	Neutral	Somewhat Satisfied	Very Satisfied
○	○	○	○	○

2. One a scale of 1 to 5, how would you rate your experience with each of the following aspects of the program? Please circle and provide any additional comments.

	Very Dissatisfied	Somewhat Dissatisfied	Neutral	Somewhat Satisfied	Very Satisfied
Culinary Demos	○	○	○	○	○
Instructor(s)	○	○	○	○	○
Weekend Hands-on Cooking	○	○	○	○	○
Instructor(s)	○	○	○	○	○
Nutrition Content	○	○	○	○	○
Instructor(s)	○	○	○	○	○
Mindfulness Content	○	○	○	○	○
Instructor(s)	○	○	○	○	○
Exercise Content	○	○	○	○	○
Instructor(s)	○	○	○	○	○
Health Coaching	○	○	○	○	○
Instructor(s)	○	○	○	○	○
