# Supplementary material for: Total Force Kitchen: Exploring Active-Duty Service Member Performance Optimization Through Cooking
Source: J Integr Complement Med. 2024 Jan 12;30(1):66–76. doi: 10.1089/jicm.2023.0025 (PMC10801678; doi:10.1089/jicm.2023.0025)
Supplement: Supplemental data [file Suppl_Data.zip › Weekly_Feedback_022217.pdf]

Subject ID: | | | | | | |

Date: | | | | | | | | |

T:________

Weekly Feedback Survey

Pilot: Teaching Kitchen at CHAMP/USO Bethesda

What surprised you?

What inspired you?

What confused you?

What would you want to learn more about?

Did you speak to your Health Coach this week? Why or why not?

If yes, how many minutes did you speak with your Health Coach this week?

Any other comments or suggestions?
