# Supplementary material for: Total Force Kitchen: Exploring Active-Duty Service Member Performance Optimization Through Cooking
Source: J Integr Complement Med. 2024 Jan 12;30(1):66–76. doi: 10.1089/jicm.2023.0025 (PMC10801678; doi:10.1089/jicm.2023.0025)
Supplement: Supplemental data [file Suppl_Data.zip › Wellbeing_Questionnaire.pdf]

Subject ID: | | | | | | |

Date: | | | | | | | | |

T:________

Well-being Assessment

Pilot: Teaching Kitchen at CHAMP/USO Bethesda

1. In general, would you say your health is:

- ○ Excellent
- ○ Very Good
- ○ Good
- ○ Fair
- ○ Poor

How TRUE or FALSE is each of the following statements for you.

	Definitely True	Mostly True	Don't Know	Mostly False	Definitely False
2. I seem to get sick a little easier than others	○	○	○	○	○
3. I am as healthy as anybody I know	○	○	○	○	○
4. I expect my health to get worse	○	○	○	○	○
5. My health is excellent	○	○	○	○	○

These questions are about how you feel and how things have been with you **during the past 4 weeks**. For each question, please give the one answer that comes closest to the way you have been feeling.

How much of the time **during the past 4 weeks** . . .

	All of the Time	Most of the Time	A Good Bit of the Time	Some of the Time	A Little of the Time	None of the Time
6. Did you feel full of pep?	○	○	○	○	○	○
7. Have you been a very nervous person?	○	○	○	○	○	○
8. Have you felt so down in the dumps that nothing could cheer you up?	○	○	○	○	○	○
9. Have you felt calm and peaceful?	○	○	○	○	○	○
10. Did you have a lot of energy?	○	○	○	○	○	○

Well-being Assessment

Pilot: Teaching Kitchen at CHAMP/USO Bethesda

	All of the Time	Most of the Time	A Good Bit of the Time	Some of the Time	A Little of the Time	None of the Time
11. Have you felt downhearted and blue?	○	○	○	○	○	○
12. Did you feel worn out?	○	○	○	○	○	○
13. Have you been a happy person?	○	○	○	○	○	○
14. Did you feel tired?	○	○	○	○	○	○
