# Supplementary material for: Total Force Kitchen: Exploring Active-Duty Service Member Performance Optimization Through Cooking
Source: J Integr Complement Med. 2024 Jan 12;30(1):66–76. doi: 10.1089/jicm.2023.0025 (PMC10801678; doi:10.1089/jicm.2023.0025)
Supplement: Supplemental data [file Suppl_Data.zip › Cooking_Nutrition (1).pdf]

Subject ID: | | | | | | |

Date: | | | | | | | | |

T:________

Cooking Nutrition Assessment

Pilot: Teaching Kitchen at CHAMP/USO Bethesda

For each item below, indicate the extent to which you agree or disagree with the statement about cooking.

	Strongly Disagree	Disagree	Neither Agree nor Disagree	Agree	Strongly Agree
1. I do NOT like to cook because it takes too much time.	○	○	○	○	○
2. Preparing meals at home would NOT improve the health of my diet.	○	○	○	○	○
3. Cooking meals is a good use of my time.	○	○	○	○	○
4. I enjoy cooking.	○	○	○	○	○
5. It is important to know how to prepare food.	○	○	○	○	○
6. Cooking is fun.	○	○	○	○	○
7. I do NOT like to prepare meals at home because it costs too much money.	○	○	○	○	○
8. It is NOT important that I know how to cook.	○	○	○	○	○
9. Cooking is interesting.	○	○	○	○	○

Cooking Nutrition Assessment

Pilot: Teaching Kitchen at CHAMP/USO Bethesda

For each item below, indicate the extent to which you agree or disagree with the statement about cooking.

	Strongly Disagree	Disagree	Neither Agree nor Disagree	Agree	Strongly Agree
10. Meals made at home are affordable.	○	○	○	○	○
11. It is important to eat the recommended 2 cups of fruit each day.	○	○	○	○	○
12. It is important to eat the recommended 2.5 cups of vegetables each day.	○	○	○	○	○
13. It is easy to prepare meals.	○	○	○	○	○
14. Cooking is frustrating	○	○	○	○	○
15. I like trying new recipes.	○	○	○	○	○
16. It is too much work to cook.	○	○	○	○	○
17. Making meals at home helps me to eat more healthfully.	○	○	○	○	○
18. I find cooking tiring.	○	○	○	○	○

Subject ID: | | | | | | |

Date: | | | | | | | | |

T:________

Cooking Nutrition Assessment

Pilot: Teaching Kitchen at CHAMP/USO Bethesda

For the 3 items below, think about your usual cooking habits.
During the past month, how often did you do the following?

	Not at all	1 to 2 times this month	Once a week	Several Times Each Week	About Everyday
19. Prepare meals from basic ingredients (such as whole fresh produce, raw chicken, etc).	○	○	○	○	○
20. Prepare meals using convenience items (such as prepared mashed potatoes.)	○	○	○	○	○
21. Reheat or use leftovers in another meal.	○	○	○	○	○

For each item below, indicate the extent to which you feel confident about performing the particular activity.

	Not at all Confident	Not Very Confident	Neither Confident nor Unconfident	Confident	Extremely Confident
22. Cook from basic ingredients (e.g., whole lettuce heads, fresh tomatoes, raw chicken)	○	○	○	○	○
23. Follow a written recipe (e.g., preparing fresh salsa from tomatoes, onion, garlic, jalapeno peppers)	○	○	○	○	○

Cooking Nutrition Assessment
Pilot: Teaching Kitchen at CHAMP/USO Bethesda

For each item below, indicate the extent to which you feel confident about performing the particular activity.

	Not at all Confident	Not Very Confident	Neither Confident nor Unconfident	Confident	Extremely Confident
24. Preparing dinner from items you currently have in your pantry and refrigerator	○	○	○	○	○
25. Using knife skills in the kitchen	○	○	○	○	○
26. Planning nutritious meals	○	○	○	○	○
27. Using basic cooking techniques	○	○	○	○	○
28. Reusing leftovers for another meal	○	○	○	○	○
29. Steaming	○	○	○	○	○
30. Sautéing	○	○	○	○	○
31. Stir-frying	○	○	○	○	○
32. Grilling	○	○	○	○	○
33. Poaching	○	○	○	○	○
34. Baking	○	○	○	○	○
35. Roasting	○	○	○	○	○
36. Stewing	○	○	○	○	○
