# Supplementary material for: Total Force Kitchen: Exploring Active-Duty Service Member Performance Optimization Through Cooking
Source: J Integr Complement Med. 2024 Jan 12;30(1):66–76. doi: 10.1089/jicm.2023.0025 (PMC10801678; doi:10.1089/jicm.2023.0025)
Supplement: Supplemental data [file Suppl_Data.zip › Follow-up Questionnaire.pdf]

ID# _______

Follow-up Questionnaire

We're interested in knowing what's been happening for you and what behaviors you've been continuing since the program's completion.

1. What changes have you personally noticed since you were involved in the Teaching Kitchens program? (i.e., physical, eating habits, shopping for food, cooking)

2. Has your participation in this program affected your choice of free-time activities with friends or family in any way? Please describe.

Culinary Skills

3. How often are you preparing meals from scratch? At home or in a shared kitchen?

4. How has this changed since the end of the program?

5. Have you been using some of the recipes you learned in class? If so, which ones?

Nutrition Information/Food Purchasing

6. Since you've learned about nutrition, what changes have you made in what you eat?

7. What changes have you made in how you shop?

8. Any other changes you've noticed?

Physical Activity

9. How many days a week do you work out/engage in physical activity?

10. What types of physical activity are you regularly following?

11. Has this increased or decreased since the end of the study?

Mind-Tactics

12. In what ways are you practicing the mind-tactics training you learned in class?

13. Have you been listening to any audio recordings or apps?

Health Coaching

14. Are you still continuing to have phone calls/emails with your health coach?

15. Were there specific goals you had discussed with your coach that you feel you are still making progress on?

16. Anything you're backsliding on?

17. Would you like to share any other comments?

Thank you for your time; your feedback is very helpful.
